# Potassium Fulvate Alleviates Salt–Alkali Stress and Promotes Comprehensive Growth of Oats in Saline–Alkali Soils of the Qaidam Basin

**DOI:** 10.3390/plants14131982

**Published:** 2025-06-28

**Authors:** Xin Jin, Jie Wang, Xinyue Liu, Jianping Chang, Caixia Li, Guangxin Lu

**Affiliations:** College of Agriculture and Animal Husbandry, Qinghai University, Xining 810016, China; 18894310895@163.com (X.J.); wangjie422022@163.com (J.W.); 17856838703@163.com (X.L.); c223663@126.com (J.C.); lcxia1314@126.com (C.L.)

**Keywords:** potassium fulvate, saline–alkali soil, rhizosphere microorganisms, soil salinization and alkalization index

## Abstract

Soil salinization limits global agricultural sustainability, and extensive areas of saline–alkaline soils on the Qinghai–Tibet Plateau remain underutilized. Against this backdrop, this study evaluated the effects and ecological regulatory mechanisms of potassium fulvate (PF) application on oat (*Avena sativa* L.) growth, soil properties, and rhizosphere microbial communities in the saline–alkali soils of the Qaidam Basin. The results showed that PF significantly enhanced both aboveground and belowground biomass and improved root morphological traits, with the higher application rate (150 kg·hm^−2^) showing superior performance. PF also effectively improved soil nutrient conditions (organic matter, ammonium nitrogen, and potassium), reduced the integrated salinity–alkalinity index, significantly optimized the composition of rhizosphere soil cations (increased K^+^ and Ca^2+^; decreased Na^+^ and Mg^2+^), and induced a marked reshaping of the composition and structure of rhizosphere microbial communities. Notably, microbial β-diversity exhibited a significant regulatory effect on the comprehensive growth of oats. Structural equation modeling (SEM) revealed that PF primarily promoted oat growth indirectly by improving soil physicochemical properties (direct effect = 0.94), while the microbial community structure served as a synergistic ecological mediator. This study clarifies the regulatory mechanisms of PF in oat cultivation under alpine saline–alkali conditions, providing both theoretical and practical support for improving soil quality, enhancing forage productivity, and promoting sustainable agriculture in cold regions.

## 1. Introduction

Globally, soil salinization is recognized as among the most pressing environmental threats to sustainable agricultural development. Salinity-driven declines in soil fertility and cultivable area have significantly compromised both food production and forage supply [1,2]. The issue is particularly severe in the arid regions of Northwest China, where high evaporation rates and low rainfall contribute to elevated soil salinity, hindering crop growth [3,4]. In the Qaidam Basin of western Qinghai Province, saline–alkali soils predominate, accounting for 98% of the province’s total salinized area [5,6]. The region is subject to severe soil salinization, characterized by high pH, poor soil structure, and low nutrient availability, all of which significantly limit land-use efficiency and agricultural productivity, thereby threatening regional economic development [7,8]. Consequently, restoring saline–alkali soils is essential not only to alleviate the shortage of cultivable land but also to improve the yield of high-quality forage in alpine regions, thereby supporting the sustainable development of local animal husbandry and integrated agroecosystems.

Among the various strategies for managing saline–alkali soils, the application of salt-tolerant crops for phytoremediation is considered both economically viable and environmentally sustainable [9]. In recent years, forage crops adapted to saline–alkaline conditions, particularly oat (*Avena sativa* L.), have been extensively cultivated in the Qaidam Basin, where multiple studies have highlighted their substantial yield potential [10,11]. However, crop selection alone is insufficient to fundamentally overcome the limitations associated with soil salinization [12]. Effective reduction of soil pH, mitigation of salt accumulation, and concurrent improvement of soil fertility represent essential prerequisites for securing high and stable crop yields [13,14]. Extensive research has demonstrated that chemical soil amendments play a pivotal role in restoring saline–alkaline soils [15]. Among the range of chemical remedies, potassium fulvate (PF) serves as an effective substitute for organic matter, enhancing soil properties, boosting crop yields, and alleviating salinity stress through both direct and indirect actions [16]. PF has also been shown to boost soil fertility, increase micronutrient availability, stimulate root growth, and enhance nutrient use efficiency, particularly under saline–alkaline stress [17,18]. Furthermore, PF modulates soil microbial communities, thereby restoring microecological functions and contributing to overall soil ecological improvement [19,20]. Although the promise of PF in saline–alkali soil remediation is well recognized, its underlying modes of action remain insufficiently understood, particularly with regard to its influence on soil attributes, salt stress, and rhizosphere microbial communities during oat cultivation in alpine saline–alkaline environments.

Based on the research background and identified knowledge gaps, this study selected saline–alkaline soil from the Delingha region, located in the Qaidam Basin of the Qinghai–Tibet Plateau, and employed the broadly adapted oat cultivar Qingtian No. 1 (*Avena sativa* L.) as the experimental plant material. Two potassium fulvate application rates (A1: 75 kg·hm^−2^ and A2: 150 kg·hm^−2^) were established to systematically evaluate the following hypotheses: (i) potassium fulvate at different application rates positively affects oat growth and overall productivity; (ii) potassium fulvate ameliorates the rhizosphere soil physicochemical properties, including reductions in salinity and alkalinity; (iii) potassium fulvate significantly influences the α- and β-diversity of rhizosphere microbial communities; and (iv) potassium fulvate directly or indirectly regulates oat growth by modulating soil nutrient availability, salinity–alkalinity stress, and microbial community structure. By rigorously testing these hypotheses, this study seeks to establish a sound scientific basis and practical recommendations for using potassium fulvate in oat cultivation within the saline and alkaline alpine zones of the Qinghai–Tibet Plateau. The findings are expected to improve land-use efficiency, strengthen ecological and economic returns, and offer new perspectives for sustainable agriculture in this fragile environment.

## 2. Materials and Methods

### 2.1. Experimental Site Description

The experimental site was located at the test field of Bensheng Forage Co., Ltd., Gahai Town, Delingha City, China, Haixi Mongolian and Tibetan Autonomous Prefecture, Qinghai Province (37°22′38.784″ N, 97°43′58.962″ E), at an altitude of 3158 m. The study area experiences an alpine climate featuring hypoxic conditions, arid air, scarce rainfall, and persistent winds. A mobile sprinkler irrigation system was available for irrigation. The previous crop was “Qingtian No. 1” oat (*Avena sativa* L.). The soil type was saline–calcic soil, with a pH ranging from 8.5 to 9.2. The basic physicochemical properties of the topsoil layer (0–30 cm) before sowing are shown in Supplementary Table S1.

### 2.2. Experimental Materials and Design

The experiment was initiated on 4 May 2023, in a representative alpine zone of the Qinghai–Tibet Plateau. The test crop was oat cultivar “Qingtian No. 1” (germination rate: 90%, purity > 90%), with a seeding rate of 300 kg·hm^−2^. Seeds were supplied by Qinghai Kairui Ecological Technology Co., Ltd. (Qinghai, China). Potassium fulvate (K_2_O: 11%, N: 4%, organic matter: 68%) was applied as the experimental amendment. Basal fertilization consisted of 300 kg·hm^−2^ of organic fertilizer and 375 kg·hm^−2^ of compound fertilizer (containing 25% total N, 12% P_2_O_5_, and 5% K_2_O), and both were incorporated into the soil prior to sowing.

**Figure 1:**
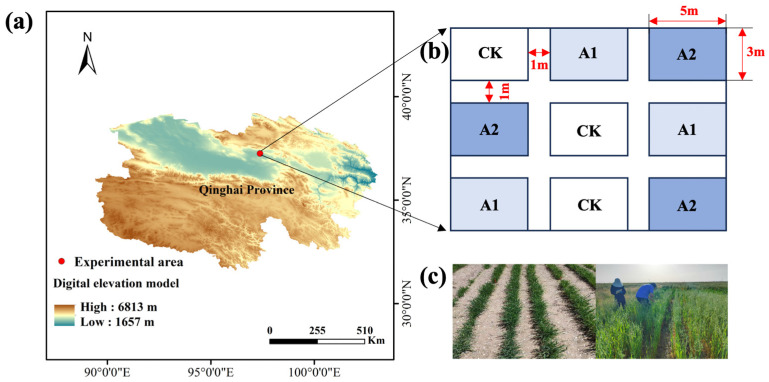
Experimental site and plot design: (**a**) experimental area, (**b**) plot design, and (**c**) field growth performance.

A completely randomized block design with three replicates per treatment was used, resulting in a total of nine plots. The treatments included no PF application (CK), 75 kg·hm^−2^ (A1), and 150 kg·hm^−2^ (A2). Each plot measured 15 m^2^ (5 m × 3 m) and was separated by 1 m buffer zones. Oats were manually sown in furrows at a depth of 3–4 cm and a row spacing of 30 cm to enhance canopy ventilation and light penetration in the semi-arid alpine environment. Potassium fulvate was applied concurrently with sowing by mixing it into the soil at the time of seed placement. The location of the experimental site and the layout of the plots are shown in Figure 1.

### 2.3. Soil and Microbial Sample Collection and Measurement

At the oat maturity stage, five sampling points were randomly selected along the diagonal of each plot. At each point, triplicate soil cores (0–15 cm depth, 38 mm in diameter) were collected with a root auger. Loosely adhering soil was removed by gentle shaking, whereas firmly attached rhizosphere soil was carefully brushed from the roots and placed in sterile bags for analyses of physicochemical traits and cation contents. Concurrently, intact root systems with attached rhizosphere soil were placed in sterile bags for microbial analysis, initially stored at −20 °C in a portable car refrigerator and subsequently transferred to a −80 °C freezer in the laboratory.

Rhizosphere soil extraction: To extract rhizosphere soil, freshly collected oat roots were placed into sterile 50 mL centrifuge tubes, and 30 mL of sterile phosphate-buffered saline (PBS, pH 7.0) containing 0.1% Tween 80 was added. The tubes were gently shaken to ensure thorough contact between the solution and root surfaces, facilitating the detachment of rhizosphere soil [21]. The samples were then ultrasonically treated for 10 min. The resulting suspension was transferred to a fresh sterile 50 mL centrifuge tube. The procedure was repeated two additional times. The pooled suspension was centrifuged at 6000 rpm for 5 min to collect the precipitate. The resulting pellet was freeze-dried using a lyophilizer at −40 °C for at least 12 h. The lyophilized rhizosphere soil was sealed and kept at −80 °C until DNA extraction.

### 2.4. Plant and Soil Measurement Indicators and Methods

Plant growth performance and root morphological traits were assessed at oat maturity (physiological maturity (panicles > 85% yellow)). In each plot, 30 uniformly growing plants were randomly selected from planting rows, and their plant heights were measured. All measurements were recorded with a precision of 0.01 cm. Six sampling segments (50 cm in length) were randomly chosen from each plot. Whole root systems were excavated, and shoots were separated by cutting at the base of the stem. Aboveground parts were used for determining fresh weight, while root systems were washed gently with low-pressure water to remove soil and used for root fresh weight measurement. Both aboveground and belowground tissues were oven-dried, initially at 105 °C for 30 min to inactivate enzymes, and then at 65 °C until a constant weight was achieved. The dry weight was subsequently recorded. For root trait analysis, 10 plants were randomly selected from each plot, and their root systems were subjected to scanning with an Epson Perfection V700 Photo scanner (Perfection V700 Photo; Seiko Epson Corporation, Suwa, Nagano, Japan). Morphological root parameters, including total root length, surface area, and root volume, were analyzed using WinRhizo Pro 2016 software (Regent Instruments Inc., Québec City, QC, Canada).

Soil physicochemical analyses: Soil pH was measured with a pH meter (PHS-3C) using a soil-to-water ratio of 1:2.5. In situ measurements of soil electrical conductivity (EC) and soil moisture content (SMC) at 0–10 cm depth were obtained with a FieldScout® TDR-350 three-parameter sensor (Spectrum Technologies Inc., Aurora, IL, USA). Total nitrogen (TN) was analyzed via the semi-micro Kjeldahl method [22], and total phosphorus (TP) was determined using the continuous flow–ammonium molybdate spectrophotometric method [23]. Organic matter (OM) was determined using the potassium dichromate–sulfuric acid external heating method [24]. Nitrate nitrogen (NO_3_^−^-N) was quantified using ultraviolet spectrophotometry [25], and ammonium nitrogen (NH_4_^+^-N) was measured using the indophenol blue colorimetric method [26]. Total potassium (TK) was determined through water bath digestion, followed by atomic fluorescence spectrometry. Potassium (K^+^) and sodium (Na^+^) ions were quantified using flame photometry, while magnesium (Mg^2+^) and calcium (Ca^2+^) ions were measured by EDTA complexometric titration [27].

### 2.5. Extraction, Amplification, and Sequence Data Processing of Rhizosphere Microorganisms

Total genomic DNA was extracted from 0.25 g of rhizosphere soil with the MoBio PowerSoil DNA Isolation Kit (QIAGEN Inc., Germantown, MD, USA). The bacterial 16S rRNA V4 region was amplified with the primers 515F/806R, whereas the fungal ITS2 region and the AMF-targeted AMF2 fragment were amplified with the primers 5.8F/4R and AMV4.5NF/AMDGR, respectively.

The 20 μL PCR reaction mixture was composed of 4 μL of 5× FastPfu Buffer, 2 μL of 2.5 mM dNTPs, 0.8 μL each of 5 μM forward and reverse primers, 0.4 μL of FastPfu DNA polymerase, and 10 ng of template DNA. PCR amplification was conducted under the following conditions: initial denaturation at 94 °C for 4 min, 25 cycles of denaturation at 94 °C for 30 s, annealing at 55 °C for 30 s, and extension at 72 °C for 1 min, followed by a final extension at 72 °C for 10 min. PCR products were confirmed on 2% agarose gels and purified using the AxyPrep DNA Gel Extraction Kit (Axygen Biosciences, Union City, CA, USA).

Purified amplicons were quantified using a Qubit 3.0 Fluorometer. Barcoded amplicons were pooled in equimolar concentrations and used to construct a paired-end 250 bp (PE250) library according to Illumina’s standard protocol. Sequencing was performed on the Illumina MiSeq platform (Shanghai LingEn Biotech Co., Shanghai, China). Raw sequences were demultiplexed based on barcode and primer sequences, and sequence orientation was adjusted. Reads shorter than 50 bp were filtered out during quality control. Paired-end reads with ≥10 bp overlap were merged with a maximum mismatch rate of 0.2, and chimeric sequences were subsequently removed. High-quality reads were denoised and clustered into amplicon sequence variants (ASVs) using the Unoise algorithm. To ensure even sequencing depth across samples, read counts were rarefied to 30,159 for 16S, 46,591 for ITS, and 24,447 for AMF datasets. The resulting ASV tables were used for downstream microbial community analyses.

### 2.6. Statistical Analysis

Before statistical analysis, all data regarding plant growth parameters, rhizosphere soil physicochemical properties, and microbial diversity indices were tested for normality and homogeneity of variance. Pairwise comparisons among treatments were performed using independent two-sample t-tests, with final *p*-values corrected by the Bonferroni method. The package “VennDiagram” was utilized to generate Venn diagrams illustrating unique and shared microbial ASVs among different treatments [28].

Differences in microbial α-diversity indices (richness, Pielou’s evenness, Shannon diversity, and phylogenetic diversity) among treatments were examined using independent two-sample t-tests, followed by Bonferroni corrections [29]. To assess the impact of potassium fulvate applications on rhizosphere microbial community structure, Bray–Curtis distance matrices were calculated with the vegan package. Non-metric multidimensional scaling (NMDS) and principal coordinate analysis (PCoA) were used to visualize the community structural variations [30]. Additionally, permutational multivariate analysis of variance (PERMANOVA) and analysis of similarity (ANOSIM) tests, with Bonferroni-adjusted *p*-values, were employed to statistically evaluate community differences among treatment groups [31]. Mantel tests were conducted to identify significant correlations between soil physicochemical variables and microbial α-diversity matrices (based on richness, Shannon, Pielou, and phylogenetic diversity indices) and β-diversity matrices (Bray–Curtis dissimilarities), visualized using the package linkET.

To further comprehensively evaluate the effects of potassium fulvate on oat growth and soil salinity–alkalinity, a comprehensive growth index (CGI) and a soil salinization and alkalization index (SSAI) were calculated. Specifically, CGI was derived by averaging the Z-scores of measured oat growth traits, including plant height, aboveground biomass, belowground biomass, root length, root surface area, and root volume. SSAI integrated key soil salinity–alkalinity parameters, including soil sodium adsorption ratio (SSAR), soil pH, and electrical conductivity (EC) [32]. The SSAR specifically represents the ratio of sodium ions to calcium and magnesium ions, with higher SSAR indicating greater risk of soil salinity and alkalinity [33].

Each component of the oat comprehensive growth and soil salinization—alkalization indices was standardized using Z-scores. Following established methods for quantifying multifunctionality in arid ecosystems [34,35], the average of Z-scores across all relevant indicators was used to calculate the comprehensive growth index (CGI) and soil salinization and alkalization index (SSAI) [36,37]. Differences in CGI and SSAI among treatments were tested for normality and variance homogeneity, followed by t-tests and Bonferroni correction for final *p*-values.

To identify primary soil and microbial factors influencing the comprehensive oat growth index (CG), random forest analysis was employed separately on soil and microbial predictor variables to avoid potential multicollinearity effects that could mask the relative importance of predictors [38,39]. Using the rfPermute package, the importance and statistical significance of each predictor were determined. Additionally, the overall model significance for selected variables was obtained using the A3 package [40]. Subsequently, critical predictors identified by random forest analysis were incorporated into a composite structural equation model (SEM) using the Piecewise SEM package [40]. The SEM examined the direct and indirect pathways through which soil and microbial factors regulated CG under potassium fulvate application. Model adequacy was confirmed by non-significant model fit tests (*p* > 0.05), and the best model was selected based on lower Akaike information criterion (AIC) and Fisher’s C values, indicating enhanced explanatory power [41]. All statistical analyses were performed using R version 4.4.1.

## 3. Results

### 3.1. Effects of Potassium Fulvate Application on Oat Growth

**Figure 2:**
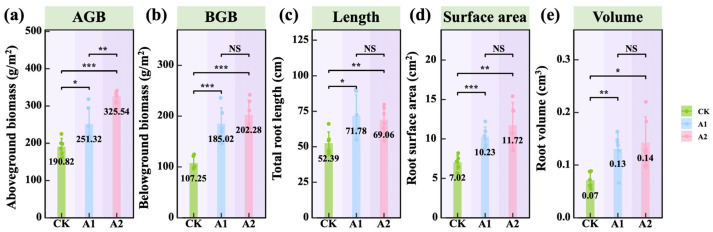
Effects of potassium fulvate on comprehensive oat growth parameters. (**a**) Aboveground biomass (AGB), (**b**) Belowground biomass (BGB), (**c**) total root length, (**d**) root surface area, and (**e**) root volume. The values in panels (**a**)–(**e**) represent means. Note: Green background in the title indicates plants. Asterisks denote statistical significance (^NS^ *p* > 0.05; * *p* < 0.05; ** *p* < 0.01; *** *p* < 0.001).

In the saline–alkali soils of Delingha, located in the Qaidam Basin on the Qinghai–Tibet Plateau, oats showed a marked growth response to potassium fulvate (PF) application. A dose-dependent increase in aboveground biomass was observed with rising PF application rates from A1 (75 kg·hm^−2^) to A2 (150 kg·hm^−2^). Both A1 and A2 treatments significantly outperformed the control (CK), with increases in aboveground biomass highly significant (*p* < 0.001; Figure 2a). Furthermore, the A2 treatment led to a significantly greater biomass than A1 (*p* < 0.01; Figure 2a). Similar trends were observed in belowground biomass, with both A1 and A2 treatments showing substantial increases compared with CK (*p* < 0.001; Figure 2b). However, no significant difference was observed between A1 and A2, indicating that the lower application rate was sufficient to effectively enhance root biomass accumulation.

Regarding root morphology, potassium fulvate (PF) application led to significant enhancements in total root length, surface area, and volume in oats. A1 treatment resulted in a significant increase in total root length compared with CK (*p* < 0.05; Figure 2c), although no further improvement was observed with the higher A2 application. Root surface area was significantly greater in both A1 and A2 treatments than in the control (*p* < 0.01; Figure 2d), and root volume exhibited a significant increase with increasing PF application rates (*p* < 0.05; Figure 2e). These findings indicate that PF application effectively promotes root system development in oats, particularly by enhancing the surface area and volume.

### 3.2. Effects of Potassium Fulvate Application on Rhizosphere Soil of Oats

**Figure 3:**
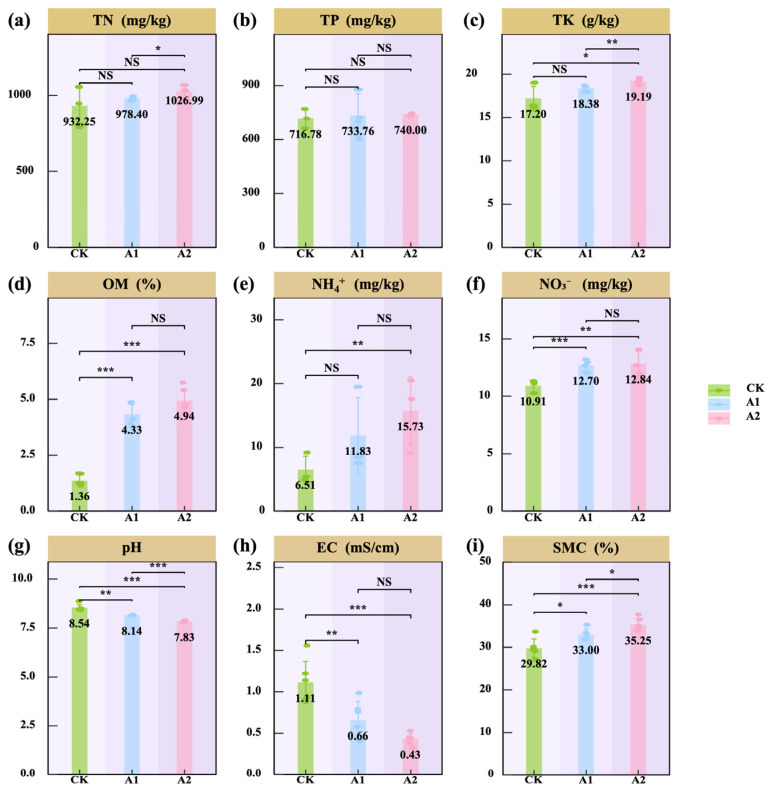
Effects of potassium fulvate on the physicochemical properties of rhizosphere soil. (**a**) Total nitrogen (TN), (**b**) total phosphorus (TP), (**c**) total potassium (TK), (**d**) organic matter (OM), (**e**) ammonium nitrogen (NH_4_^+^), (**f**) nitrate nitrogen (NO_3_^−^), (**g**) soil pH (pH), (**h**) electrical conductivity (EC), and (**i**) soil moisture content (SMC). The values in panels (**a**–**i**) represent means. Note: Brown background in the title indicates plants. Asterisks denote statistical significance (^NS^ *p* > 0.05; * *p* < 0.05; ** *p* < 0.01; *** *p* < 0.001).

The physicochemical properties of the oat rhizosphere soil were improved appreciably following PF application. TN content increased significantly with higher PF application rates, and A2 was significantly higher than the control (CK) (*p* < 0.05; Figure 3a). In contrast, TP content remained unaffected by PF (*p* > 0.05; Figure 3b). PF application also enhanced potassium availability, as TP content was significantly higher in both A1 and A2 treatments compared with CK (*p* < 0.05; Figure 3c). Nitrogen availability and related indices were substantially elevated by PF application, including increases in OM, NH_4_^+^, and NO_3_^−^ levels. Among these, OM content increased significantly (*p* < 0.001; Figure 3d), and nitrate nitrogen (NO_3_^−^) also increased significantly (*p* < 0.01; Figure 3f), while ammonium nitrogen (NH_4_^+^) accumulation was most pronounced under the A2 treatment (*p* < 0.01; Figure 3e). Soil pH was modestly reduced under PF treatments, with both A1 and A2 showing significantly lower values than CK (*p* < 0.01; Figure 3g), indicating a mild acidification effect. Electrical conductivity (EC) also decreased significantly (*p* < 0.01; Figure 3h), suggesting an alleviation of salt stress. In addition, soil moisture content (SMC) increased significantly under both A1 and A2 treatments (*p* < 0.05; Figure 3i), indicating enhanced soil water-holding capacity.

**Figure 4:**
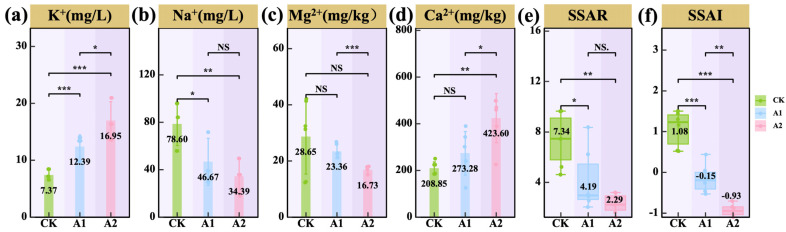
Effects of potassium fulvate on cations of rhizosphere soil. (**a**) Rhizospheric soil K^+^, (**b**) rhizospheric soil Na^+^, (**c**) rhizospheric soil Mg^2+^, (**d**) rhizospheric soil Ca^2+^, (**e**) soil sodium adsorption ratio (SSAR), and (**f**) soil salinization and alkalization index (SSAI). Note: Brown background in the title indicates rhizosphere soil. Asterisks denote statistical significance (^NS^ *p* > 0.05; * *p* < 0.05; ** *p* < 0.01; *** *p* < 0.001).

Potassium fulvate (PF) application altered the composition of major cations in the oat rhizosphere soil. PF significantly increased K^+^ concentrations in both A1 and A2 compared with the control (CK) (*p* < 0.001; Figure 4a), with A2 also exceeding A1 (*p* < 0.05), indicating that higher PF input further promoted potassium accumulation. In contrast, Na^+^ concentrations decreased significantly in both A1 and A2 compared with CK (*p* < 0.05; Figure 4b). Mg^2+^ levels declined progressively with increasing PF rates, with a highly significant reduction observed in A2 compared with CK (*p* < 0.001; Figure 4c). In contrast, Ca^2+^ concentrations increased significantly under PF application, with both A1 and A2 higher than CK and A2 significantly exceeding A1 (*p* < 0.05; Figure 4d). Furthermore, both the soil sodium adsorption ratio (SSAR) and the soil salinization and alkalization index (SSAI) declined with increasing PF application. Both A1 and A2 significantly reduced SSAR and SSAI (*p* < 0.05; Figure 4e,f), especially under the A2 treatment, indicating that PF application mitigates soil salinity and alkalinity stress. In summary, potassium fulvate application effectively modulated the cation composition in saline–alkali rhizosphere soil by increasing K^+^ and Ca^2+^ concentrations while reducing Na^+^ and Mg^2+^ levels, thereby alleviating salt stress. Among all treatments, the high application rate (A2) exerted the strongest regulatory effect.

### 3.3. Effects of Potassium Fulvate on the Composition of Rhizosphere Soil Microbial Communities

Applying potassium fulvate (PF) reshaped the structure of both unique and shared amplicon sequence variants (ASVs) within oat rhizosphere microbial communities. Although a substantial proportion of bacterial ASVs was conserved across all treatments (Supplementary Figure S1a), both A1 and A2 treatments harbored a considerable number of unique taxa, suggesting that PF selectively enriched specific bacterial groups while preserving the core microbiome. Fungal communities exhibited a pronounced dose-dependent shift in response to PF application. A1 retained more ASVs in common with the control (CK), whereas the number of unique ASVs in A2 was lower (Supplementary Figure S1b), suggesting potential community turnover or suppression at higher application rates. In contrast, arbuscular mycorrhizal fungal (AMF) communities exhibited greater compositional turnover, with fewer shared ASVs among treatments (Supplementary Figure S1c), indicating that AMF were more sensitive to PF.

Comparative analyses at both the phylum and genus levels were performed to assess the effects of potassium fulvate (PF) on rhizosphere microbial composition. At the phylum level, Proteobacteria, Actinobacteriota, and Bacteroidota were identified as the dominant phyla within the bacterial communities (Supplementary Figure S2a). The relative abundance of Proteobacteria increased in PF-treated groups compared with the control (CK). Actinobacteriota exhibited a decreasing trend with increasing PF application (A1 > CK > A2), whereas Bacteroidota displayed the opposite pattern (CK < A1 < A2). For fungi, Ascomycota and Chytridiomycota were the dominant phyla (Supplementary Figure S2c). The relative abundance of Ascomycota increased with PF application (A2 > A1 > CK), while Chytridiomycota exhibited the opposite trend (CK > A2 > A1). The AMF community was dominated by Glomeromycota (Supplementary Figure S2e), whose relative abundance decreased progressively with increasing PF dosage (CK > A1 > A2). At the genus level, *Pseudomonas* and *Sphingomonas* dominated the bacterial community, whose relative abundances declined progressively with increasing PF application rates (CK > A1 > A2; Supplementary Figure S2b). *Fusarium*, *Penicillium*, and *Scytalidium* dominated the fungal community (Supplementary Figure S2d). The relative abundances of *Fusarium* and *Penicillium* reached their highest levels under the A2 treatment (A2 > A1 > CK), while *Scytalidium* showed the highest abundance under A1 (A1 > CK > A2). For AMF, *Paraglomus* and *Glomus* constituted the main genera (Supplementary Figure S2f). *Paraglomus* showed a decreasing trend (CK > A1 > A2), in contrast with *Glomus*, which increased with PF application (A2 > A1 > CK).

### 3.4. Effects of Potassium Fulvate on Rhizosphere Microbial Diversity and Community

**Figure 5:**
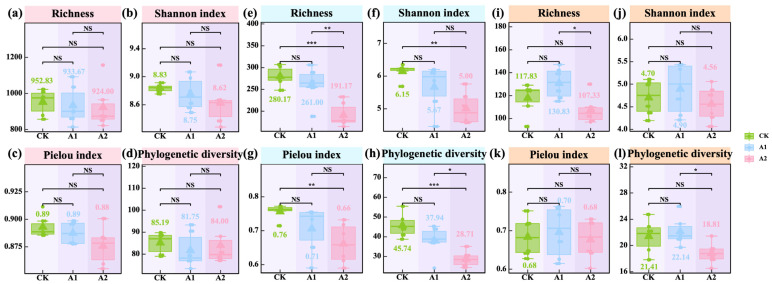
Effects of potassium fulvate on the α-diversity of rhizosphere microbial communities. (**a**–**d**) represent rhizosphere soil bacterial communities, (**e**–**h**) represent rhizosphere soil fungal communities, and (**i**–**l**) represent rhizosphere arbuscular mycorrhizal fungal (AMF) communities. The values in panels (**a**–**l**) represent means. Note: Pink background in the title indicates rhizosphere soil bacteria, blue background indicates rhizosphere soil fungi, and orange background indicates rhizosphere arbuscular mycorrhizal fungi (AMF). Asterisks denote statistical significance (^NS^ *p* > 0.05; * *p* < 0.05; ** *p* < 0.01; *** *p* < 0.001).

Rhizosphere microbial diversity responded differentially to potassium fulvate (PF) application, depending on community type. In the bacterial community, richness, Pielou’s evenness, Shannon diversity, and phylogenetic diversity remained statistically unchanged (*p* > 0.05; Figure 5a–d), indicating bacterial community stability under exogenous PF input. In contrast, fungal communities responded more strongly to PF application, as both A1 and A2 treatments significantly reduced richness (*p* < 0.05; Figure 5e). The high application rate (A2) led to significant reductions in fungal Shannon diversity and phylogenetic diversity (*p* < 0.05; Figure 5f,h), whereas Pielou’s evenness was not significantly affected. In AMF communities, richness and phylogenetic diversity declined significantly under the A2 treatment (*p* < 0.05; Figure 5i,l), while Shannon diversity and Pielou’s evenness remained stable (*p* > 0.05; Figure 5j,k).

**Figure 6:**
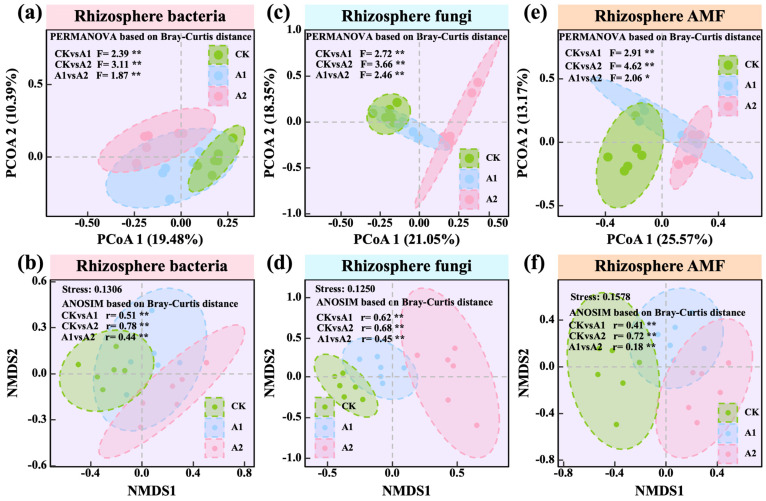
Effects of potassium fulvate on rhizosphere microbial communities (based on principal coordinate analysis (PCoA) and non-metric multidimensional scaling (NMDS)). (**a**,**b**) Rhizosphere soil bacterial community, (**c**,**d**) rhizosphere soil fungal community, (**e**,**f**) rhizosphere arbuscular mycorrhizal fungal (AMF) community. Note: Pink background in the title indicates rhizosphere soil bacteria, blue background indicates rhizosphere soil fungi, and orange background indicates rhizosphere arbuscular mycorrhizal fungi (AMF). Asterisks denote statistical significance (* *p* < 0.05; ** *p* < 0.01).

Potassium fulvate (PF) application induced gradual shifts in the structure of oat rhizosphere microbial communities, with stronger effects observed at higher application rates. Principal coordinate analysis (PCoA) revealed distinct clustering of CK, A1 (75 kg·hm^−2^), and A2 (150 kg·hm^−2^) in bacterial, fungal, and AMF communities. In the bacterial community, PCoA1 explained 19.48% of the total variation. PERMANOVA results confirmed significant differences among treatments (CK vs. A1: F = 2.39; CK vs. A2: F = 3.11; A1 vs. A2: F = 1.87; all *p* < 0.01; Figure 6a). The fungal community exhibited more distinct clustering, with PCoA1 explaining 21.05% of the variation and all pairwise comparisons highly significant (*p* < 0.01; Figure 6c). The AMF community was the most responsive to PF application, with PCoA1 explaining 25.57% of the variation and the greatest dissimilarity observed between CK and A2 (F = 4.62; *p* < 0.01; Figure 6e). NMDS and ANOSIM analyses corroborated these findings. Bacterial communities were clearly differentiated between CK and both A1 and A2, as well as between A1 and A2 (*p* < 0.01; Figure 6b). In fungal communities, all treatment groups exhibited distinct separation (*p* < 0.01; Figure 6d). Despite relatively smaller effect sizes, the AMF community still displayed significant separation among all treatments (*p* < 0.01; Figure 6f).

Mantel test results revealed significant shifts in the relationships between rhizosphere microbial diversity and soil physicochemical properties under potassium fulvate (PF) application. Microbial responses to environmental variables differed across α- and β-diversity metrics.

**Figure 7:**
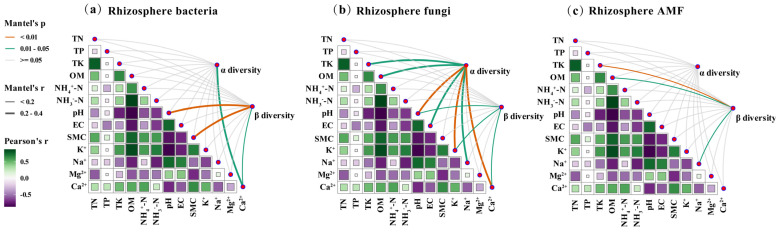
Mantel test analysis of the correlation between bacterial and fungal diversity (α and β) and rhizospheric soil properties. (**a**) Rhizosphere soil bacterial community, (**b**) rhizosphere soil fungal community, and (**c**) rhizosphere arbuscular mycorrhizal fungal (AMF) community. Note: Detailed Mantel test statistics for the correlations between microbial diversity indices and soil variables are provided in Supplementary Tables S2–S4.

Among bacterial communities, only Ca^2+^ was significantly positively correlated with α-diversity (r = 0.222, *p* = 0.029; Figure 7a, Supplementary Table S2), whereas other soil variables were not significantly associated. Bacterial β-diversity was positively associated with soil pH (r = 0.209, *p* = 0.005), SMC (SMC; r = 0.202, *p* = 0.007), and Ca^+^ (r = 0.174, *p* = 0.013; Figure 7a, Supplementary Table S2), suggesting that bacterial community composition was shaped by spatial variation in alkalinity, moisture, and calcium availability. In fungal communities, α-diversity exhibited significant positive correlations with multiple soil variables (Figure 7b, Supplementary Table S3), including TK (r = 0.206, *p* = 0.04), OM (r = 0.212, *p* = 0.03), pH (r = 0.367, *p* = 0.004), EC (r = 0.215, *p* = 0.026), K^+^ (r = 0.340, *p* = 0.002), Na^+^ (r = 0.211, *p* = 0.025), and Ca^2+^ (r = 0.251, *p* = 0.01), with pH, K^+^, and Ca^2+^ being the strongest predictors. Fungal β-diversity was also positively correlated with pH (r = 0.183, *p* = 0.014), K^+^ (r = 0.170, *p* = 0.025), and Ca^2+^ (r = 0.166, *p* = 0.021), highlighting the role of ion-associated variables in shaping fungal community structure. In contrast, AMF α-diversity showed no significant associations with any soil variables, indicating that species richness was relatively stable across soil conditions. However, AMF β-diversity was significantly correlated with TK (r = 0.179, *p* = 0.002), OM (r = 0.145, *p* = 0.016), and Na^+^ (r = 0.184, *p* = 0.017; Figure 7c, Supplementary Table S4), reflecting community-level sensitivity to nutrient and salinity gradients, particularly potassium and sodium ions.

### 3.5. Effects and Regulatory Pathways of Potassium Fulvate on the Comprehensive Growth of Oats

**Figure 8:**
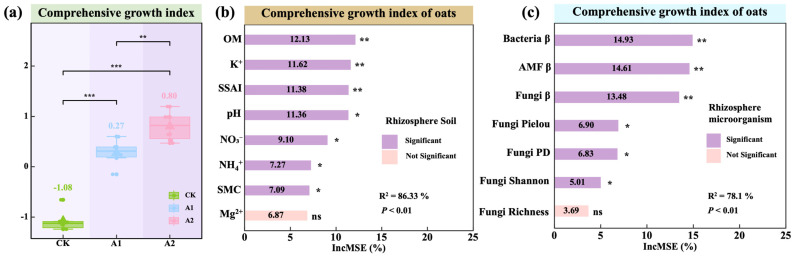
Differences in the comprehensive growth index of oat and the relative contributions of key driving factors. (**a**) Variations in the oat comprehensive growth index. The values in figure (**a**) indicate means. (**b**) Relative contributions of rhizosphere soil key factors to changes in the comprehensive growth index. (**c**) Relative contributions of rhizosphere microorganism key factors to changes in the comprehensive growth index. Note: (**b**,**c**) are based on random forest analysis and show the top 8 and top 7 most influential variables, respectively, selected from a large set of predictors. Note: Green background in the title indicates plants, brown background in the title indicates rhizosphere soil, and blue background indicates rhizosphere microorganism (^ns^ *p* > 0.05; * *p* < 0.05; ** *p* < 0.01; *** *p* < 0.001).

A comprehensive growth index (CGI) was developed to quantify overall oat performance based on plant height, aboveground biomass, belowground biomass, root length, root surface area, and root volume. CGI was significantly higher under A1 and A2 treatments compared with the control (CK) (*p* < 0.001; Figure 8a), with A2 significantly surpassing A1 (*p* < 0.01), indicating a stronger growth-promoting effect at higher PF application rates.

A random forest model was used to identify the most important predictors of CGI variation (Figure 8b). In the soil domain, organic matter (12.13%), potassium (K^+^; 11.62%), the soil salinization and alkalization index (SSAI; 11.38%), and pH (11.36%) were significant contributors to CGI (*p* < 0.05). Nitrate nitrogen (NO_3_^−^), ammonium nitrogen (NH_4_^+^), and soil moisture content (SMC) also contributed significantly (*p* < 0.05; Figure 8b). In the microbial domain, bacterial β-diversity (14.93%), AMF β-diversity (14.61%), and fungal β-diversity (13.48%) were identified as the most influential ecological predictors (*p* < 0.01; Figure 8c). Fungal Pielou’s evenness, phylogenetic diversity, and Shannon index were also significantly associated with CGI (*p* < 0.05), while fungal richness was not statistically significant. These results suggest that PF enhances comprehensive oat growth through the coordinated regulation of rhizosphere nutrient availability and microbial community structure, with microbial β-diversity acting as a primary ecological driver.

**Figure 9:**
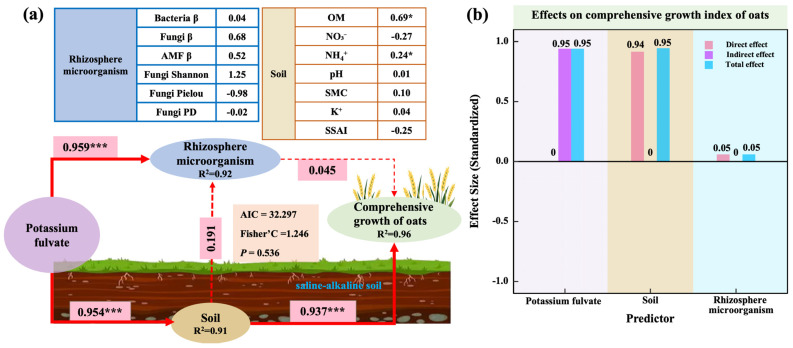
Regulatory pathways influencing comprehensive oat growth performance. (**a**) Structural equation modeling (SEM) reveals the regulatory pathways. (**b**) Quantitative contributions of potassium fulvate, soil properties, and microbial communities to the comprehensive growth index, including their direct, indirect, and total effects. Note: Red lines indicate positive effects, with line thickness reflecting the strength of the effect (* *p* < 0.05; *** *p* < 0.001).

A structural equation model (SEM) was constructed to elucidate the regulatory pathways by which potassium fulvate (PF) influences the comprehensive growth index (CGI). The final model showed excellent fit (AIC = 32.297; Fisher’s C = 1.246; *p* = 0.536; Figure 9a), explaining 96% of the variation in CGI (R^2^ = 0.96). The effect of PF on oat growth was fully mediated through indirect pathways, with a standardized total effect of 0.95 (*p* < 0.001; Figure 9b).

Specifically, PF significantly improved soil properties (β = 0.954; *p* < 0.001; Figure 9a), which in turn strongly promoted CGI (β = 0.937; *p* < 0.01; Figure 9a). PF also had a significant positive effect on the rhizosphere microbial community (β = 0.950; *p* < 0.001; Figure 9a). However, its direct influence on CGI was limited (β = 0.045; *p* > 0.05; Figure 9a). Effect decomposition revealed that soil exerted both a direct and total effect of 0.94 on CGI, whereas the microbial community contributed an indirect effect of 0.05 (Figure 9b).

In terms of individual predictors (Figure 9a), organic matter (OM; β = 0.69) and ammonium nitrogen (NH_4_^+^; β = 0.24) had significant positive effects (*p* < 0.05), whereas NO_3_^−^ (β = −0.27) and the soil salinization and alkalization index (SSAI; β = −0.25) emerged as key negative predictors. Among microbial indicators, AMF β-diversity (β = 0.68) showed a strong positive association with CGI, while fungal Shannon diversity (β = −1.25) and Pielou’s evenness (β = −0.98) were negatively associated, underscoring the greater importance of community compositional structure over α-diversity metrics. While some variables did not reach statistical significance within the linear SEM framework, this does not preclude their ecological relevance. As revealed by the random forest results (Figure 8b,c), these variables still played important roles in a nonlinear context, suggesting potentially complex relationships with CGI.

Overall, PF enhanced the comprehensive growth index (CGI) of oats by optimizing rhizosphere nutrient availability and soil physicochemical conditions, while concurrently reshaping microbial community structure. Among these, soil factors served as the primary drivers, with microbial communities functioning as secondary but important modulators.

## 4. Discussion

### 4.1. Potassium Fulvate Application Promotes Aboveground and Belowground Growth of Oat

This study confirms that potassium fulvate (PF) application significantly promotes the growth of oat in saline–alkaline soils of the Qinghai–Tibet Plateau, particularly through enhanced biomass accumulation and improved root system architecture. Comparable growth-enhancing effects of PF and analogous humic amendments have been reported in barley and wheat subjected to similar salinity stress [42,43]. As a natural organic acid compound, PF exhibits strong metal-chelating capacity and controlled ion-release behavior, which collectively mitigate nutrient antagonism and osmotic stress under saline–alkali conditions. These effects improve the rhizosphere environment and enhance plant uptake of water and essential mineral nutrients [16,17,18]. PF application significantly enhanced oat aboveground biomass, with a more pronounced effect observed at higher application rates. The high application rate (A2) conferred a significant yield advantage over the lower rate (A1), suggesting that, under pronounced saline–alkali stress, increased PF input can further enhance shoot productivity [16].

Belowground responses revealed that even the lower application rate (A1) effectively promoted root biomass accumulation and structural development. This suggests that PF at low dosages can facilitate root differentiation, elongation, and thickening. Increases in total root length and surface area expand the absorptive interface, thereby improving the efficiency of water and nutrient acquisition and supporting aboveground growth [44,45]. Notably, no significant differences were observed between A1 and A2 in root biomass and most root morphological parameters, suggesting a possible saturation effect of PF on root development. Excessive application may result in diminishing returns and increased risks of resource wastage or ion accumulation in soil. Comparable plateaus have been reported in other cereals: in rice, root growth stimulation by fulvic acid leveled off beyond a certain threshold [46]; in wheat, high doses of humic substances produced no further root development benefits [47].

### 4.2. Potassium Fulvate Enhances Nutrient Availability, Balances Ions, and Alleviates Saline–Alkali Stress in the Rhizosphere

Under saline–alkali soil conditions, potassium fulvate (PF) application significantly enhanced nutrient availability and ion balance in the oat rhizosphere and effectively alleviated soil salinization and alkalization. These regulatory effects were dose dependent, with the most pronounced responses observed under the high application rate. Similar trends have been reported in barley and wheat, where fulvate or humic substances improved nutrient uptake and ionic regulation under salt stress, particularly at higher doses [42,48].

To begin with, PF substantially increased the concentrations of total nitrogen, total potassium, and their available forms (ammonium and nitrate nitrogen) in rhizosphere soil, with a particularly significant rise in organic matter content. These improvements are likely attributable to the abundance of reactive organic functional groups in PF, which (i) stimulate microbial activity and accelerate organic matter mineralization, and (ii) chelate nutrient ions, thereby prolonging their retention and enhancing bioavailability [16]. Similar nutrient enhancement effects under saline conditions have been reported in wheat and maize following humic or fulvic acid treatments [49,50]. In contrast, total phosphorus levels remained largely unchanged, suggesting a limited impact of PF on phosphorus availability and highlighting the potential benefit of combining PF with phosphate fertilizers in future applications.

PF also enhanced soil physicochemical properties. Reductions in soil pH and electrical conductivity suggest partial acidification and desalination, likely mediated by weakly acidic moieties (e.g., carboxyl groups) that neutralize alkaline ions and promote Na^+^ exchange and leaching [51,52]. The observed increase in soil moisture content indicates improved soil aggregation and moisture-holding capacity, thereby creating a more favorable environment for root activity [53]. To improve cation balance and reduce saline–alkali stress, PF application significantly increased K^+^ and Ca^2+^ concentrations while reducing Na^+^ and Mg^2+^ levels. These changes optimized rhizosphere ionic composition, maintained cellular ion homeostasis, and enhanced plant salt tolerance. These findings are consistent with earlier reports on potassium humate enhancing salt tolerance in alfalfa under saline conditions [54]. PF also significantly lowered the soil sodium adsorption ratio (SSAR) and the soil salinization and alkalization index (SSAI), consistent with reductions in pH, EC, and Na^+^ levels in our study.

### 4.3. Potassium Fulvate Alters Rhizosphere Microbial Community Composition

At the amplicon sequence variant (ASV) level, PF application enriched specific microbial lineages while maintaining a core set of shared ASVs within the bacterial community. Both A1 and A2 treatments fostered distinct bacterial assemblages, suggesting that PF enhances functional diversification without compromising the stability of the core microbiome [19]. In contrast, the fungal community exhibited greater sensitivity to PF dosage: A1 retained a substantial number of ASVs shared with the control, while A2 showed a marked reduction in unique ASVs, indicating potential taxonomic replacement or functional loss under high-dose application. The arbuscular mycorrhizal fungal (AMF) community underwent pronounced species turnover, as evidenced by the low proportion of shared ASVs across treatments, highlighting their heightened sensitivity to PF input. Previous studies have likewise demonstrated that the combined amendment of humic acid and AMF substantially restructures soil microbial communities, inducing significant shifts in AMF composition [55].

At the taxonomic level, the relative abundances of Proteobacteria and Bacteroidota significantly increased following PF application, suggesting key ecological roles in salt stress mitigation and organic matter decomposition. These shifts align with observed improvements in rhizosphere salinity and alkalinity under PF treatment. Within fungi, Ascomycota exhibited a dose-dependent increase, with genera such as Fusarium and Penicillium significantly enriched under PF treatment, suggesting that PF promotes the proliferation of functional or antagonistic taxa. These findings are consistent with previous reports highlighting PF-induced enhancement of fungal activity and its role in nitrogen cycling and microbial functional diversity [56]. AMF responded selectively to PF application, with *Glomus* exhibiting increased abundance, suggesting that PF may enhance rhizosphere symbiotic networks and promote more efficient nutrient uptake by host roots [57].

### 4.4. Potassium Fulvate Reshapes Rhizosphere Microbial Diversity and Underlying Environmental Drivers

We evaluated the regulatory effects of potassium fulvate application on rhizosphere microbial diversity and community structures in saline–alkaline soils, revealing distinct community-type specificity, dosage-dependent responses, and associations with key environmental factors.

At the diversity level, differential responses among microbial taxa to potassium fulvate application were observed. Bacterial diversity metrics, including richness, evenness (Pielou’s index), Shannon diversity, and phylogenetic diversity, remained relatively stable across treatments, suggesting resilience or stability in bacterial communities following potassium fulvate supplementation [58]. In contrast, fungal and arbuscular mycorrhizal fungal (AMF) communities exhibited greater sensitivity to potassium fulvate treatments. Specifically, under the higher application rate (A2), fungal communities showed significant declines in both Shannon diversity and phylogenetic diversity, whereas AMF communities maintained overall diversity but exhibited a marked reduction in species richness. Parallel observations in tobacco rhizospheres indicate that potassium fulvate amendments likewise drive profound restructuring of fungal communities, underscoring the remarkable ecological plasticity of eukaryotic soil microorganisms [59]. These findings suggest that eukaryotic microbial communities respond more dramatically to potassium fulvate [58], potentially due to their distinct trophic strategies, symbiotic lifestyles, and differential dependency on rhizosphere resource availability.

Regarding community structure, the concordant results obtained from PCoA, NMDS, and ANOSIM analyses indicated significant restructuring of bacterial, fungal, and AMF communities following potassium fulvate application, with the magnitude of structural differentiation increasing alongside application rate increments. Such dosage-dependent structural reshaping aligns well with prior research [58]. Notably, AMF communities exhibited the most pronounced structural differentiation under the highest potassium fulvate dose, confirming a clear dose-dependent sensitivity. Independent investigations of potassium humate and fulvate biostimulants have likewise reported marked alterations in AMF structure and function under elevated application rates, especially when plants face abiotic stress [46,47].

Further insights from Mantel tests elucidated distinct response pathways linking microbial communities to soil physicochemical factors. Bacterial community β-diversity was primarily driven by soil pH, soil moisture content (SMC), and Ca^2+^ concentrations, reflecting their spatial responsiveness to variations in alkalinity and soil moisture conditions. Comparable observations in forest ecosystems indicate that bacterial assemblages are likewise regulated by gradients in pH, calcium concentration, and soil moisture [60]. In contrast, both α- and β-diversities of fungal communities showed heightened sensitivity to multiple soil factors, including soil organic matter, total potassium, electrical conductivity, K^+^, Na^+^, and Ca^2+^. Among these, the regulatory roles of pH, K^+^, and Ca^2+^ were especially pronounced, highlighting fungal communities’ robust ecological feedback to ionic environment shifts [58]. AMF α-diversity appeared relatively stable across soil environments; however, their community structure (β-diversity) significantly correlated with total potassium, organic matter content, and sodium concentration, suggesting that AMF community assembly processes are governed by the interactive effects of nutrient availability and salinity conditions [61].

### 4.5. Effects of Potassium Fulvate on Comprehensive Growth Index and Regulatory Pathways

A comprehensive growth index (CGI) was developed to quantify PF-induced improvements in overall oat growth by integrating plant height, aboveground biomass, belowground biomass, root length, root surface area, and root volume. Both low (A1: 75 kg·hm^−2^) and high (A2: 150 kg·hm^−2^) PF application rates significantly improved CGI, with A2 showing a greater effect. Similar improvements have been reported where fulvic acids enhanced soil aggregate stability and humus quality, humic acid fertilizer blends boosted cotton productivity, and humic amendments lowered salinity while optimizing rhizosphere conditions during sodic soil reclamation [62,63]. Such dosage-dependent effects are consistent with prior findings on the role of humic substances in alleviating physicochemical stress and improving rhizosphere conditions in saline–alkali soils [16].

Integrated random forest and structural equation modeling (SEM) analyses revealed two complementary regulatory pathways through which PF promoted growth: (i) direct modification of soil physicochemical properties and (ii) indirect regulation via microbial community restructuring. Soil organic matter (OM), potassium (K^+^), pH, and salinity were identified as key soil drivers, reflecting PF’s capacity to increase nutrient availability and reduce ionic toxicity [16,54]. Microbial β-diversity—particularly that of AMF—emerged as a major predictor of CGI. This finding aligns with observed microbial restructuring under PF treatment, with AMF β-diversity contributing 14.61% to CGI, highlighting that spatial variability in functional guilds better predicts plant growth than α-diversity indices. Similar patterns have been reported across grassland ecosystems, where plant and soil microbial β-diversity more accurately predicted above- and belowground biomass than α-diversity metrics, emphasizing the ecological importance of community turnover in driving productivity [64].

SEM further confirmed that the soil pathway was the primary positive driver (direct effect = 0.94). PF enhanced CGI through increased OM and ammonium nitrogen (NH_4_^+^) and reductions in nitrate nitrogen (NO_3_^−^) and the soil salinization and alkalization index (SSAI). While the microbial pathway had a weaker direct effect (β = 0.045), it still played an essential ecological role within a nonlinear framework, potentially through feedback regulation and functional redundancy that enhance plant stress resilience. Comparable mechanistic partitioning has been demonstrated: structural equation modeling indicates that, under salinity, soil organic matter and moisture enhance crop yield indirectly by reshaping the microbial community, although direct soil-mediated effects remain the predominant driver [65]. Interestingly, fungal Shannon and Pielou’s evenness indices were negatively correlated with CGI, suggesting that, under saline conditions, higher fungal α-diversity may not benefit plant growth [66]. Instead, the stability of core fungal taxa better supports plant adaptation under stress [67].

Our findings indicate that potassium fulvate improves oat performance chiefly by ameliorating soil physicochemical conditions and, in parallel, by enhancing the rhizosphere β-diversity of bacteria, fungi, and arbuscular mycorrhizal fungi. This synergistic soil–microbe pathway provides a quantitative framework for optimizing oat cultivation on saline–alkaline soils. The results underscore the importance of prioritizing soil remediation, complemented by targeted manipulation of rhizosphere community composition, to advance sustainable agricultural practices in salt-affected landscapes.

## 5. Conclusions

Our study demonstrates that potassium fulvate markedly enhances the overall growth of oat on saline–alkaline soil. The underlying mechanism combines improvements in soil physicochemical properties, including higher organic matter, greater available potassium, lower pH, and reduced salinity, with changes in rhizosphere microbial communities, as shown by the increased β-diversity of bacterial, fungal, and arbuscular mycorrhizal fungal (AMF) populations. Structural equation modeling revealed a dominant direct soil pathway, whereas the microbial pathway, though exerting a limited direct effect, provided critical ecological regulation through nonlinear feedbacks. Among the treatments tested, applying potassium fulvate at 150 kg·hm^−2^ produced the greatest growth response. These results confirm potassium fulvate as an efficient organic amendment for alleviating soil salinization and increasing forage yield, providing both theoretical understanding and practical recommendations for managing saline–alkaline soils and boosting productivity in the cold, high-altitude Qaidam Basin.

## Data Availability

Data is deposited in the National Microbiology Data Center (NMDC) with accession numbers NMDC10019863 (https://nmdc.cn/resource/genomics/project/detail/NMDC10019863).

## Supplementary Materials

The following supporting information can be downloaded at https://www.mdpi.com/article/10.3390/plants14131982/s1. Figure S1. The effect of potassium fulvate on the species composition of rhizosphere soil microbial communities. Figure S2. The effect of potassium fulvate on the composition of rhizosphere soil microbial communities. Table S1. Basic physicochemical properties of topsoil (plow layer soil). Table S2. Mantel test results for the correlations between bacterial diversity and rhizosphere soil. Table S3. Mantel test results for the correlations between fungi diversity and rhizosphere soil. Table S4. Mantel test results for the correlations between arbuscular mycorrhizal fungal (AMF) diversity and rhizosphere soil.
