# Supplementary material for: Potassium Fulvate Alleviates Salt–Alkali Stress and Promotes Comprehensive Growth of Oats in Saline–Alkali Soils of the Qaidam Basin
Source: Plants (Basel). 2025 Jun 28;14(13):1982. doi: 10.3390/plants14131982 (PMC12251709; doi:10.3390/plants14131982)
Supplement: Supplementary file 1 [file plants-14-01982-s001.zip › plants-3686105-supplementary.pdf]

Supplementary materials for

Potassium Fulvate Alleviates Salt–Alkali Stress and Promotes Comprehensive Growth of Oats in Saline–Alkali Soils of the Qaidam Basin

Xin Jin †, Jie Wang †, Xinyue Liu, Jianping Chang, Caixia Li, Guangxin Lu *

College of Agriculture and Animal Husbandry, Qinghai University, Xining 810016, China; 18894310895@163.com (X.J.); wangjie422022@163.com (J.W.) 17856838703@163.com (X.L.); c223663@126.com (J.C); lcxia1314@126.com (C.L.)

* Correspondence: lugx74@163.com; Tel.: +86-13897216290

† These authors contributed equally to this work.

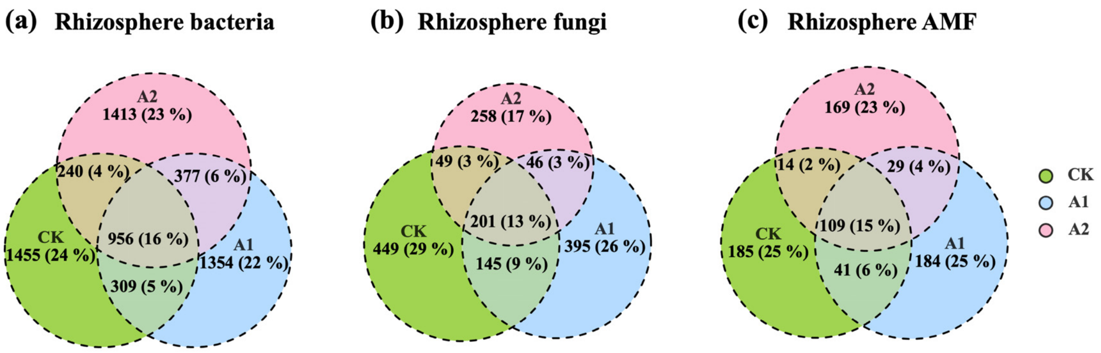

Figure S1. The effect of Potassium Fulvate on the species composition of rhizosphere soil microbial communities.

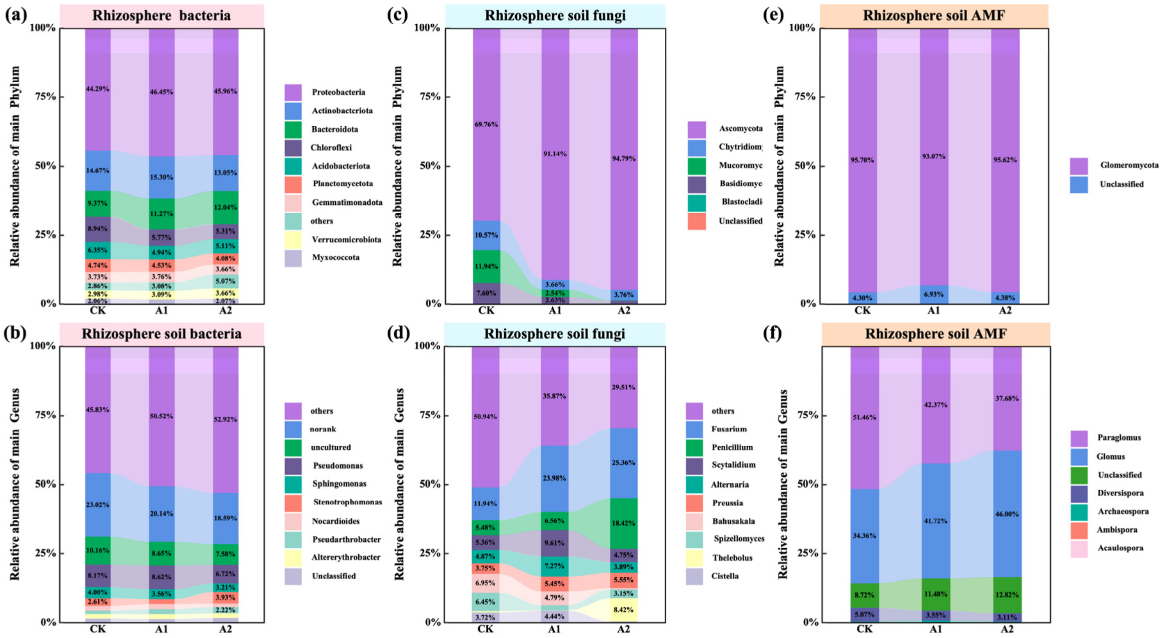

Figure S2. The effect of Potassium Fulvate on the composition of rhizosphere soil microbial communities

Table S1. Basic physicochemical properties of topsoil (plough layer soil).

Soil physicochemical properties	Content
pH	9.05
Soil organic matter / %	1.05
Nitrate nitrogen / ($mg \cdot kg^{-1}$)	10.21
Ammonium nitrogen / ($mg \cdot kg^{-1}$)	4.28
Total phosphorus / ($mg \cdot kg^{-1}$)	639
Total nitrogen / ($mg \cdot kg^{-1}$)	739
Total potassium / ($g \cdot kg^{-1}$)	14.17

Table S2. Mantel test results for the correlations between bacterial diversity and rhizosphere soil.

Diversity	Soil	r	*p*	sign
α_diversity	TN	0.013	0.388	ns
α_diversity	TP	-0.122	0.724	ns
α_diversity	TK	0.123	0.153	ns
α_diversity	OM	0.084	0.172	ns
α_diversity	NH_4^+-N	-0.06908	0.713	ns
α_diversity	NO_3^--N	0.076	0.245	ns
α_diversity	pH	0.14	0.158	ns
α_diversity	EC	0.141	0.163	ns
α_diversity	SMC	0.054	0.314	ns
α_diversity	K^+	-0.008	0.48	ns
α_diversity	Na^+	0.194	0.059	ns
α_diversity	Mg^{2+}	-0.079	0.657	ns
α_diversity	Ca^{2+}	0.222	0.029	*
β_diversity	TN	0.004	0.441	ns
β_diversity	TP	-0.066	0.806	ns
β_diversity	TK	0.105	0.122	ns
β_diversity	OM	0.081	0.167	ns
β_diversity	NH_4^+-N	0.033	0.39	ns
β_diversity	NO_3^--N	0.002	0.458	ns
β_diversity	pH	0.209	0.005	**
β_diversity	EC	0.073	0.181	ns
β_diversity	SMC	0.202	0.007	**
β_diversity	K^+	0.021	0.364	ns
β_diversity	Na^+	0.075	0.185	ns
β_diversity	Mg^{2+}	-0.021	0.564	ns
β_diversity	Ca^{2+}	0.174	0.013	*

Asterisks indicate the level of significance ($^{ns}p > 0.05$; $*p < 0.05$; $**p < 0.01$).

Table S3. Mantel test results for the correlations between fungi diversity and rhizosphere soil.

Diversity	Soil	r	*p*	sign
α_diversity	TN	0.091	0.187	ns
α_diversity	TP	-0.177	0.969	ns
α_diversity	TK	0.206	0.04	*
α_diversity	OM	0.212	0.03	*
α_diversity	NH_4^+-N	0.009	0.353	ns
α_diversity	NO_3^--N	0.179	0.051	ns
α_diversity	pH	0.367	0.004	**
α_diversity	EC	0.215	0.026	*
α_diversity	SMC	0.139	0.093	ns
α_diversity	K^+	0.34	0.002	**
α_diversity	Na^+	0.211	0.025	*
α_diversity	Mg^{2+}	0.041	0.288	ns
α_diversity	Ca^{2+}	0.251	0.01	**
β_diversity	TN	-0.074	0.862	ns
β_diversity	TP	-0.099	0.929	ns
β_diversity	TK	0.091	0.126	ns
β_diversity	OM	0.071	0.21	ns
β_diversity	NH_4^+-N	0.035	0.331	ns
β_diversity	NO_3^--N	0.047	0.272	ns
β_diversity	pH	0.183	0.014	*
β_diversity	EC	0.078	0.167	ns
β_diversity	SMC	-0.01	0.534	ns
β_diversity	K^+	0.17	0.025	*
β_diversity	Na^+	0.133	0.07	ns
β_diversity	Mg^{2+}	-0.058	0.744	ns
β_diversity	Ca^{2+}	0.166	0.021	*

Asterisks indicate the level of significance ($^{ns}p > 0.05$; $^{*}p < 0.05$; $^{**}p < 0.01$).

Table S4. Mantel test results for the correlations between arbuscular mycorrhizal fungal (AMF) diversity and rhizosphere soil.

Diversity	Soil	r	*p*	sign
α_diversity	TN	0.044	0.34	ns
α_diversity	TP	0.079	0.268	ns
α_diversity	TK	0.014	0.387	ns
α_diversity	OM	-0.05	0.664	ns
α_diversity	NH_4^+-N	0.026	0.324	ns
α_diversity	NO_3^--N	-0.028	0.591	ns
α_diversity	pH	0.029	0.352	ns
α_diversity	EC	-0.067	0.651	ns
α_diversity	SMC	-0.119	0.858	ns
α_diversity	K^+	0.089	0.151	ns
α_diversity	Na^+	-0.043	0.626	ns
α_diversity	Mg^{2+}	-0.013	0.496	ns
α_diversity	Ca^{2+}	0.063	0.222	ns
β_diversity	TN	0.077	0.203	ns
β_diversity	TP	-0.095	0.923	ns
β_diversity	TK	0.179	0.002	**
β_diversity	OM	0.145	0.016	*
β_diversity	NH_4^+-N	-0.078	0.78	ns
β_diversity	NO_3^--N	0.127	0.075	ns
β_diversity	pH	0.104	0.109	ns
β_diversity	EC	0.098	0.113	ns
β_diversity	SMC	-0.057	0.723	ns
β_diversity	K^+	0.065	0.196	ns
β_diversity	Na^+	0.184	0.017	*
β_diversity	Mg^{2+}	-0.042	0.628	ns
β_diversity	Ca^{2+}	0.073	0.238	ns

Asterisks indicate the level of significance ($^{ns}p > 0.05$; $^{*}p < 0.05$; $^{**}p < 0.01$).
